# Nut Consumption and Fertility: a Systematic Review and Meta-Analysis

**DOI:** 10.1016/j.advnut.2023.100153

**Published:** 2023-11-17

**Authors:** Barbara R. Cardoso, Izabella Fratezzi, Nicole J. Kellow

**Affiliations:** 1 Department of Nutrition, Dietetics and Food, Monash University, Victoria, Australia; 2 Victorian Heart Institute, Monash University, Clayton, Victoria, Australia; 3 Centre for Innate Immunity and Infectious Diseases, Hudson Institute of Medical Research, Clayton, Victoria, Australia

**Keywords:** nuts, diet, fertility, male infertility, female infertility, sperm quality

## Abstract

The high concentration of omega-3 polyunsaturated fats, dietary fibers, vitamins, minerals, and polyphenols found in nuts suggest their regular consumption may be a simple strategy for improving reproductive health. This systematic review and meta-analysis aimed to present up-to-date evidence regarding the association between nut intake and fertility outcomes in males and females. Ovid MEDLINE, Embase, CINAHL, and Scopus were searched from inception to 30 June 2023. Eligible articles were interventional or observational studies in human subjects of reproductive age (18–49 y) that assessed the effects (or association) of dietary nut consumption (for a minimum of 3 mo) on fertility-related outcomes. Random-effects meta-analyses were completed to produce a pooled effect estimate of nut consumption on sperm total motility, vitality, morphology, and concentration in healthy males. Four studies involving 875 participants (646 males, 229 females) were included in this review. Meta-analysis of 2 RCTs involving 223 healthy males indicated consumption of ≥ 60g nuts/d increased sperm motility, vitality, and morphology in comparison to controls but had no effect on sperm concentration. Nonrandomized studies reported no association between dietary nut intake and conventional sperm parameters in males, embryo implantation, clinical pregnancy or live birth in males and females undergoing ART. Our meta-analysis shows that including at least 2 servings of nuts daily as part of a Western-style diet in healthy males improves sperm parameters, which are predictors of male fertility. Due to their nutritional profile, nuts were found to have potential to promote successful reproductive outcomes.

This trial was registered at PROSPERO (CRD42020204586).

**Statement of Significance**

This first-of-its-kind systematic review and meta-analysis shows that daily consumption of at least 2 servings of nuts (60 g) daily improves semen quality in healthy males. We discuss the main potential mechanisms involved in such benefits and point directions for future research.

## Introduction

Infertility is a condition characterized by the failure to achieve a clinical pregnancy after 12 mo of regular and unprotected sexual intercourse [1]. Infertility affects multiple areas of a couple’s life and can have devastating long-term social, psychological, and financial consequences [2]. It is estimated that between 8 and 12% of reproductive-aged couples in the world are affected by infertility [1]. Furthermore, infertility in females of reproductive age has been estimated to affect 1 in every 7 couples in Western countries and 1 in every 4 couples in developing countries, reaching rates as high as 30% [3]. Male infertility rates are reported to be highest in Africa and Central/Eastern Europe, whereas rates for North America, Australia, and Central and Eastern Europe vary from 4.5 to 12% [4].

Common factors that negatively affect a couple’s ability to successfully conceive a child include advanced maternal and/or paternal age, malnutrition, endocrine disorders such as obesity or polycystic ovarian syndrome (PCOS), complications associated with untreated sexually transmitted infections (STIs), and sperm production abnormalities. However, in 10 to 15% of cases, the etiology of infertility remains unknown [5]. Although assisted reproductive technologies (ART) are a common treatment option for subfertility, not all affected couples can afford it. The average cost of a standard in vitro fertilization (IVF) cycle was estimated at U$19,200 in the United States [6], although this amount can significantly vary across countries [7]. Considerable economic, racial, ethnic, geographic, and cultural disparities in access to fertility treatments also exist [8]; thus, research aiming at maximizing natural fertility is of utmost importance.

Emerging scientific efforts focus on identifying modifiable factors that affect fertility, such as diet and other health behaviors. The literature in this field has greatly expanded over the last decade, recognizing some dietary patterns associated with higher reproductive potential. The most studied dietary pattern regarding fertility is the Mediterranean diet (MedDiet), which is characterized by the high intake of mono and polyunsaturated fats from fish and olive oil, and high consumption of fruits, vegetables, whole grains, legumes, and nuts, and moderate alcohol consumption [9]. Observational prospective cohort studies have demonstrated that adherence to this diet has been associated with a larger number of embryos available, fertilized oocytes, and embryo yield in infertile females undergoing IVF treatment [10], as well as increased probability of pregnancy in females undergoing IVF/intracytoplasmic sperm injection (ICSI) treatment [[11], [12], [13]]. In males, evidence from cross-sectional studies shows that MedDiet consumption is associated with increased sperm concentration, total sperm count, and total and progressive motility [14, 15]. Further, Gaskins et al. [16] designed the Pro-Fertility diet based on foods and nutrients previously related to ART outcomes aiming to maximize females’s fertility. Despite some similarities with the MedDiet, the Pro-Fertility diet emphasizes the importance of specific nutrients such as folic acid, B12, and vitamin D, necessary for optimal fetal development, as well as the low exposure to pesticides by prioritizing low-pesticide fruits and vegetables. In an observational study, they found that high adherence to this dietary pattern was associated with higher probability of implantation, clinical pregnancy, and live birth, whereas no associations were observed for estradiol trigger levels, endometrial thickness, total or mature oocyte yield, or number of embryos [16]. Nonetheless, observational studies report conflicting evidence regarding the association between adherence to the Pro-Fertility diet and markers of ovarian reserve [17, 18], demonstrating the complexity of the modifiable factors on fertility.

Although the studies that aim to establish a relationship between dietary patterns and fertility outcomes can provide insightful information, they may also represent a challenge to those who have very distinct dietary habits. Thus, identifying the benefits of particular foods can elucidate dietary strategies that are easier to implement. Nuts can be a strong ally for fertility management given their nutritional profile, characterized by a high ratio of omega-3: omega-6 fatty acids, low concentration of saturated fats, and large concentration of proteins, fibers, vitamins, minerals, and bioactive compounds with potential redox action [19]. The benefits of nut intake have been associated with reduced risk of different chronic diseases such as diabetes [[20], [21], [22]], cognitive impairment [23], and cardiovascular diseases [24, 25]. Given the importance of diet as a modifiable factor to reduce infertility, this systematic review and meta-analysis aims to present up-to-date evidence regarding the role of nutrition in fertility, focusing on nuts as a key component to improve reproductive health. We further discuss the potential mechanisms regulated by nuts that are involved in fertility.

## Methods

### Study identification

The aim of this review was to synthesize the outcomes of published human studies investigating the effect of dietary nut consumption on fertility. This review was conducted in accordance with the PRISMA statement [26] and was prospectively registered on a systematic literature review registration website (PROSPERO, Registration No. CRD42020204586). Research literature databases Ovid MEDLINE, Scopus, CINAHL, and Embase were searched from database inception to 30 June 2023. The PICOS framework used was Population: males and females of reproductive age (for the purpose of this review, “reproductive age” was defined as adults aged 18–49 y); Intervention: Nut consumption; Comparator: no or low nut consumption; Outcome: biochemical fertility markers, clinical pregnancy or live birth, Study design: all study types (cross-sectional, case-control, cohort, and RCTs). Studies were included if published in either English or Spanish and involved human participants. All databases were searched using the terms: Adult∗ AND (Diet OR Nuts OR “Prunus Dulcis” OR Almond∗ OR Anacardium OR Cashew∗ OR Corylus OR Hazelnut∗ OR Pistacia OR Pistachio∗ OR Juglans OR Walnut∗ OR Carya OR Pecan∗ OR Arachis OR Peanut∗ OR Pinus OR ‘Pine nut∗’ OR Bertholletia OR ‘Brazil nut∗’) AND (Pregnancy OR ‘Live Birth’ OR Fertility OR Infertility OR Fecundity OR ‘Sperm quality’ OR ‘Semen quality’ OR ‘Sperm concentration’ OR ‘Sperm motility’ OR ‘Sperm DNA fragmentation’ OR embryo∗ OR ‘embryo morphology’ OR oocyte∗). The full search strategy is presented in Supplemental Table 1. Studies with a cross-sectional, case-control, cohort, or clinical trial study design, which recruited human subjects of reproductive age, assessed dietary nut consumption for a minimum of 3 mo, and measured any fertility-related outcomes were eligible for inclusion. Duration of nut consumption for a minimum of 3 mo was chosen as a criterion for inclusion in this review as it takes up to 76 d (2.5 mo) for sperm cell maturation [27], and therefore nut consumption was required to be maintained during this timeframe. Excluded trials involved human participants outside of reproductive age (<18 y or >49 y), did not quantify dietary nut intake, only quantified dietary nut intake when grouped together with other foods (e.g., seeds, beans, soy products) or assessed nut consumption for less than 3 mo, were animal, ecological or in vitro studies, or measured outcomes which were unrelated to fertility. Reference lists of selected studies and reviews were manually searched to supplement the electronic search.

### Screening and eligibility

All resultant references were imported into a systematic review screening and data extraction software program (Covidence Systematic Review Software, Veritas Health Innovation), which was used to screen studies and identify those meeting the prespecified inclusion criteria. The Covidence program automatically identified and eliminated duplicate articles. During the first pass, article titles and abstracts were screened by 2 of the listed authors (IF, NJK, BRC) independently to determine their suitability for inclusion. Selected studies then underwent full-text screening, which was also conducted by 2 of the listed authors (IF, NJK, BRC) independently. Conflicts were resolved by discussion until consensus was reached. On completion of screening, the PRISMA Flowchart was automatically generated by the Covidence program.

### Risk of bias assessment and data extraction

Risk of bias in eligible studies was independently assessed by 2 separate authors (NJK and BRC) using either the Cochrane Risk of Bias 2 tool for randomized trials (RoB 2) [28] or risk of Bias in Nonrandomized Studies–of Interventions (ROBINS-I) tool [29]. The RoB 2 tool identifies potential sources of bias within RCTs based on a set of 5 domains, including bias arising from the randomization process, deviations from intended interventions, incomplete outcome data, outcome measurement bias, and selective reporting of results. Risk of bias for each RCT was designated as either “low risk,” “some concerns,” or “high risk.” Studies that were assigned as “low risk” for all 5 items were considered to have an overall low risk of bias; studies assigned “some concerns” for ≥1 items (in the absence of any “high risk” items) were considered to have some concerns regarding their risk of bias, whereas studies allocated ≥1 “high risk” items were designated as having a high risk of bias overall. The ROBINS-I tool identifies potential biases within nonrandomized studies based on a set of 7 domains, including confounding, selection of participants, classification of interventions, deviations from intended interventions, incomplete outcome data, outcome measurement bias, and selective reporting of results. Risk of bias for each study was classified as either “no information,” “low risk,” “moderate risk,” “serious risk,” or “critical risk.” Studies assigned “moderate risk” for ≥1 items (in the absence of any “serious risk” or “critical risk” items) were considered to have an overall moderate risk of bias, whereas studies allocated ≥1 “serious risk” items were designated as having a serious risk of bias overall. Inconsistencies between the authors' risk of bias assessments at the study level were resolved through active discussion until consensus was reached.

Upon completion of screening and risk of bias assessments, data were independently extracted from each article by all authors using a data collection table. Data collected included first author, year of publication, country in which the trial was conducted, mean age of participants (y), gender of participants (female or male), mean body mass index (BMI) of participants (kg/m^2^), type of nuts consumed, quantity of nuts consumed, methodology used to assess nut intake, length of the intervention/study, rate of follow-up and the fertility outcomes measured.

### Statistical analysis

A meta-analysis was carried out for the outcomes available in the RCTs: sperm total motility, sperm morphology, sperm vitality, and sperm concentration. Semen quality outcomes were subjected to a random-effects model meta-analyses using Review Manager (RevMan) (Version 5.1. Copenhagen: The Nordic Cochrane Centre, The Cochrane Collaboration, 2014). Using change data for control and intervention groups, the standardized mean difference (SMD) was determined for each outcome with 95% confidence intervals (CIs). SMD values of 0.2, 0.5, and 0.8 were considered to represent small, moderate, and large effect sizes, respectively [30]. Where the manuscript provided median and IQR values, the mean and standard deviation were estimated using the method of McGrath (2020) [31]. Results were combined for each outcome, and data were tested for interstudy heterogeneity using the Cochrane Q statistic and quantified by the *I*^2^ statistic with *P* < 0.10. An I^2^ > 50% was considered substantial heterogeneity.

## Results

### Study selection

**FIGURE 1:**
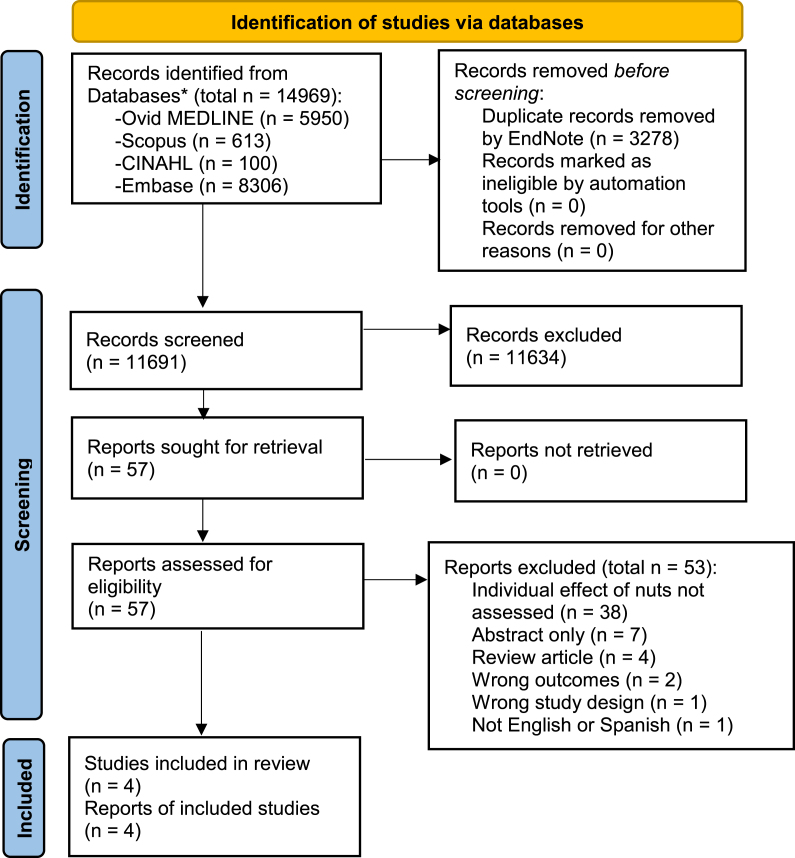
PRISMA flowchart detailing progression of studies through the review process. ∗ Hand searching of the reference lists of the included studies was undertaken, but no additional studies were identified.

The initial search resulted in 11,691 articles (after removal of duplicates), of which 11,634 were excluded upon initial screening for not meeting inclusion criteria. The remaining 57 papers were comprehensively assessed for eligibility, with 53 being excluded for the following reasons: not human trials, not available in English or Spanish, did not include a target outcome, not a relevant intervention, and insufficient detail of dietary intervention. All studies screened specified a dietary intake assessment timeframe, and no studies were found that collected dietary intake information for a duration of < 3 mo. During the title, abstract, and full-text screening phases, no conflicts between reviewers occurred (interrater agreement was 100%). Four studies involving 875 (646 males, 229 females) participants fulfilled the inclusion criteria and were included in the systematic review, and 2 of the 4 studies were included in the meta-analysis (Figure 1).

### Study characteristics

Two of the studies included in this review were RCTs with parallel dietary interventions, collectively involving 223 healthy males. Both studies targeted males within a similar age range (overall 18–35 y) who reported consuming a typical Western-style diet. Robbins et al. [32] provided 75 g of whole-shelled English walnuts/d for 12 wk as the intervention, whereas Salas-Huetos et al. [33] provided 60 g nuts (30 g walnuts, 15 g almonds, 15 g hazelnuts) to their experimental group for a period of 14 wk. In both studies, the control group continued consuming a regular diet without nuts. The primary outcomes for both studies included conventional semen parameters (concentration, vitality, motility, and morphology). Robbins et al. [32] further assessed sperm aneuploidy as part of the primary outcomes, and the secondary outcomes included sperm fatty acid concentrations and blood hormones (total testosterone, estradiol, follicle stimulating hormone [FSH], luteinizing hormone [LH], and sex hormone binding globulin [SHBG]). The study by Salas-Huetos et al. [33] further included sperm concentration and pH as primary outcomes and assessed sperm reactive oxygen species (ROS) production, DNA fragmentation, methylation, chromosome stability, and microRNA expression as secondary outcomes. A cohort study included in this review aimed to examine the association between dietary nut intake and fertility outcomes in 229 couples undergoing ARTs [34]. Dietary intake of peanuts, walnuts, and other nuts were quantified individually and combined to estimate “total nut intake” using a validated 131-item food frequency questionnaire (FFQ). The primary outcomes assessed were the associations between both maternal and paternal dietary nut intake and probability of embryo implantation, clinical pregnancy, and live birth. Secondary outcomes included associations between maternal nut intake and probability of pregnancy loss and the relationship between male nut consumption and conventional markers of semen quality. A case-control study comparing the dietary intakes of 40 males with subfertility **(**oligoasthenoteratozoospermia**)** and 40 males with normozoospermia also met the criteria for inclusion in this review [35]. The authors estimated dietary nut consumption using interviews and an FFQ (validation status unknown). The characteristics of the 4 studies included in the present systematic review are shown in Table 1.

**TABLE 1:** Nut consumption and fertility outcomes in males and females of reproductive age

First author (year of publication), country of origin	Trial methodology, length of intervention	Number of participants, gender, mean age (y), mean participant BMI (kg/m^2^)	Type & quantity of nuts consumed	Methods used to assess dietary intake and monitor nut intake	Study completion rate or % follow-up	Outcomes measured	Effect of nut consumption versus comparator on fertility outcomes
Robbins (2012), USA [32]	Parallel RCT, single-blinded, 12 wk	117 healthy males consuming a Western-style diet, mean age: 25y (range: 21–35y), mean BMI: 25 kg/m^2^	75g walnuts/d (intervention) versus no nuts	Diet history questionnaire and 3-d food record, random telephone 24-hr recalls twice monthly to assess nut intake compliance (outcome not stated)	n = 117 randomized, n = 117 provided baseline data, n = 112 completed the study (96%)	Sperm: - concentration - total motility - morphology - vitality - aneuploidy Sperm & Serum: - ALA - DHA - omega-6 - omega-3 Sex Hormones: - FSH - Testosterone	Sperm: ↔concentration ↑total motility ↑morphology ↑vitality ↔aneuploidy Serum: ↑ALA ↔DHA ↑omega-6 ↑omega-3 Sex Hormones: ↑FSH ↔Testosterone
Salas-Huetos (2018), Spain [33]	Parallel RCT, blinding not stated, 14 wk	119 healthy males consuming a Western-style diet, mean age: 24.5y (range: 18–35y), mean BMI: 24 kg/m^2^	60g mixed nuts/d (intervention) versus no nuts	3-d food record, return of empty nut sachets to assess nut intake compliance (>95%)	n = 119 randomized, n = 106 provided baseline data, n = 98 completed the study (82%)	Sperm: - count - concentration - total motility - morphology - vitality - aneuploidy - DNA fragmentation - DNA methylation - microRNA expression - ROS	Sperm: ↑count ↔concentration ↑total motility ↑morphology ↑vitality ↔aneuploidy ↓DNA fragmentation ↔DNA methylation ↓microRNA expression ↔ROS
Salas-Huetos (2022), USA [34]	Prospective cohort, 2007–2020	229 females and their male partners receiving ART, median age: 35y (IQR 32–38) (females) and 36y (IQR 33.6–39.3) (males), median BMI: 23 kg/m^2^ (IQR 21–25.7) (females) and 27 kg/m^2^ (IQR 24.3–28.9) (males). 343 males contributed to semen analysis data	Median (IQR) total nut intake: 0.4 (0.2, 0.7) serves/d (females). (0.4 serves nuts = approx. 12g)	Validated 131-item FFQ	N/A	Primary outcomes: - embryo implantation - clinical pregnancy - live birth Secondary outcomes: - total pregnancy loss (females) - semen volume, sperm count, sperm concentration, sperm total and progressive motility, sperm morphology (males)	Association between intake of total nuts, peanuts, walnuts and other nuts (males and females) and primary outcomes: ↔ embryo implantation ↔ clinical pregnancy ↔ live birth Association between intake of total nuts, peanuts, walnuts and other nuts (females) and secondary outcomes: ↔ total pregnancy loss Association between intake of total nuts, peanuts, walnuts and other nuts (males) and secondary outcomes: ↔ semen volume, sperm count, sperm concentration, sperm total and progressive motility, sperm morphology
Yorusun (2020), Turkey [35]	Case-control	40 males with subfertility (low semen volume, sperm concentration, sperm count, sperm motility and sperm progressive motility according to WHO criteria) and 40 males with normozoospermia, mean age: 34.7 ± 5.6y (subfertile group) and 34.7 ± 6.0y (normozoospermic group) (*P* > 0.05), mean BMI: 28.2 ± 4.0 kg/m^2^ (subfertile group) and 26.6 ± 3.0 kg/m^2^ (normozoospermic group) (*P <* 0.05)	Median (IQR) nut intake: 8 (26) g/d (subfertile group), 12 (34.8) g/d (normozoospermic group) (*P =* 0.99)	Interview and FFQ (type and validation of FFQ not stated)	N/A	Diet: - Dietary nut intake/d Sperm: - sperm concentration - sperm count - sperm motility - sperm progressive motility - sperm rapid motility	Diet: ↔ dietary nut intake/d between groups Sperm: Significant positive correlation between nut intake/d and sperm motility (rho=0.234, *P <* 0.05) and sperm progressive motility (rho= 0.269, *P <* 0.05) (total group)

Abbreviations: ART: assisted reproductive technology; ALA: alpha-linolenic acid; BMI: body mass index; FFQ: food frequency questionnaire; FSH: follicle stimulating hormone; IQR: interquartile range; RCT: randomized controlled trial; ROS: reactive oxygen species; WHO: World Health Organization; ↓: significantly lower than in the comparison control group after intervention; ↑: significantly higher than in the comparison control group after intervention; ↔: no significant difference between the nut-supplemented and control groups after intervention.

### Risk of Bias

**FIGURE 2:**
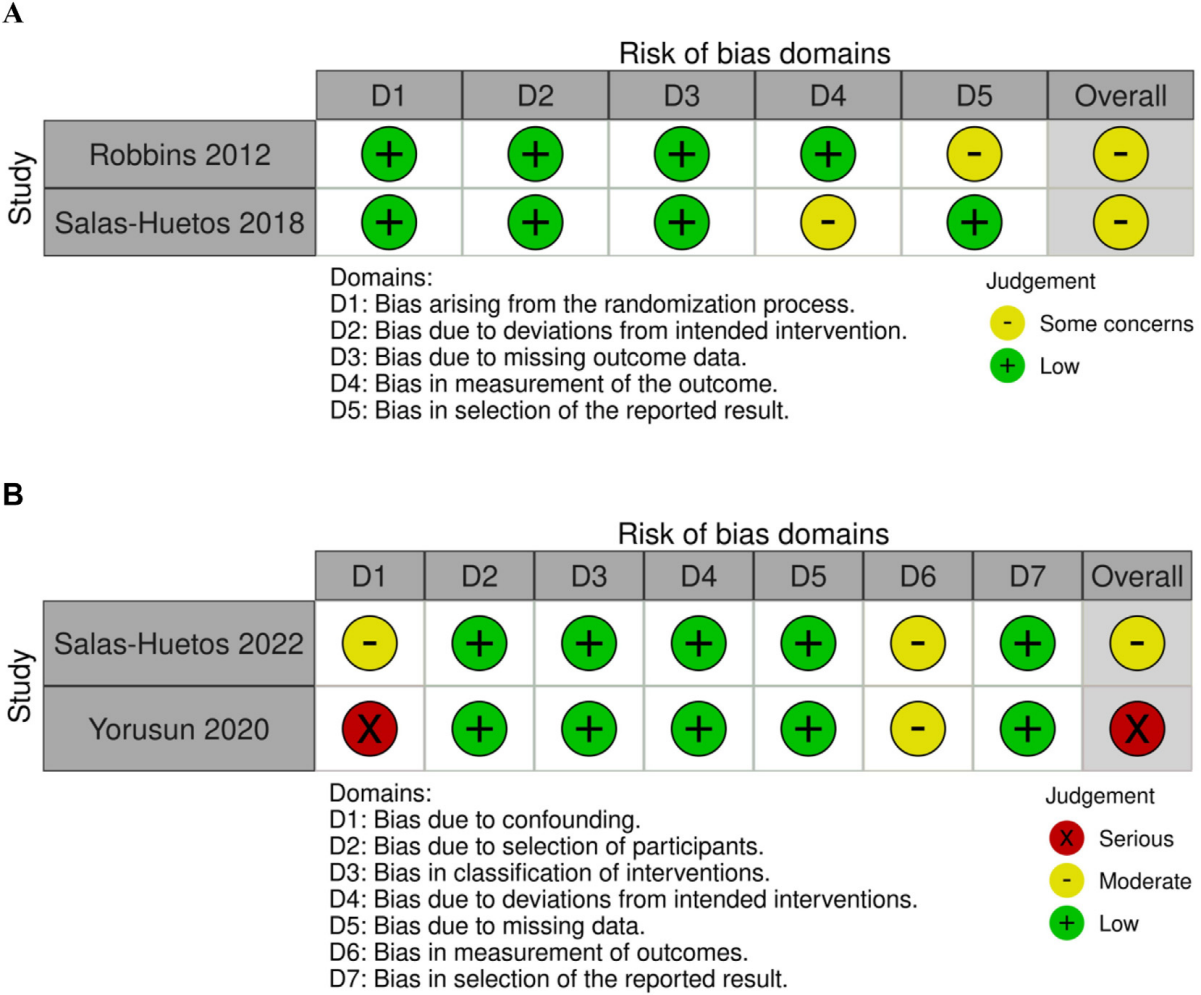
Risk of bias results for included studies using the A) Cochrane Risk of Bias 2 (RoB 2) tool and B) Risk of Bias in Nonrandomized Studies–of Interventions (ROBINS-I) tool (traffic light plot).

Both RCTs included in this review were determined to have “some concerns” regarding their risk of bias according to the Cochrane RoB 2 tool (Figure 2A). The main sources of bias included failure to disclose whether outcome assessors or statisticians were blinded [33] and failure to prepublish a statistical analysis plan in a clinical trials registry [32]. Of the 2 nonrandomized studies, one was determined to have a moderate risk of bias [34], and the other had a serious risk of bias [35] according to the ROBINS-I tool (Figure 2B). The main sources of bias in these studies included failure to adjust for major confounders [35] and failure to disclose whether outcome assessors or statisticians were blinded [34, 35].

### Outcomes

Salas-Huetos et al. [34] found no association between dietary intake of total nuts, peanuts, walnuts, or other nuts and probability of implantation, clinical pregnancy, or live birth in 229 females or their male partners following ART. There was also no association between maternal nut intake and pregnancy loss. Nut consumption was rather low in this cohort, with the females in the highest quartile of nut intake only consuming a median of 0.9 servings of total nuts/day. Similarly, the authors found no association between dietary nut intake and conventional semen parameters in males, although the association between walnut intake and total sperm count approached significance (*P* trend = 0.05). Interestingly, the same study found significant associations between DHA and EPA consumption and increased probability of live birth in females. Dietary ALA intake in males was also positively associated with sperm count and concentration. In their case-control study, Yorusun and colleagues [35] reported that although males with subfertility had a significantly higher BMI and consumed more sugar-sweetened beverages, alcohol, and meals outside of home than their fertile counterparts, there was no significant difference in nut intake between groups (*P* = 0.992). However, dietary nut intake was very low overall, with a median nut consumption of 8 and 12 grams per day in the subfertile and normozoospermic groups, respectively, the equivalent of ≤0.4 servings of nuts per day. The authors found a small but significant positive correlation between nut intake and sperm motility and progressive motility, but these results are questionable, considering that the statistical analysis was not adjusted for key confounders.

**FIGURE 3:**
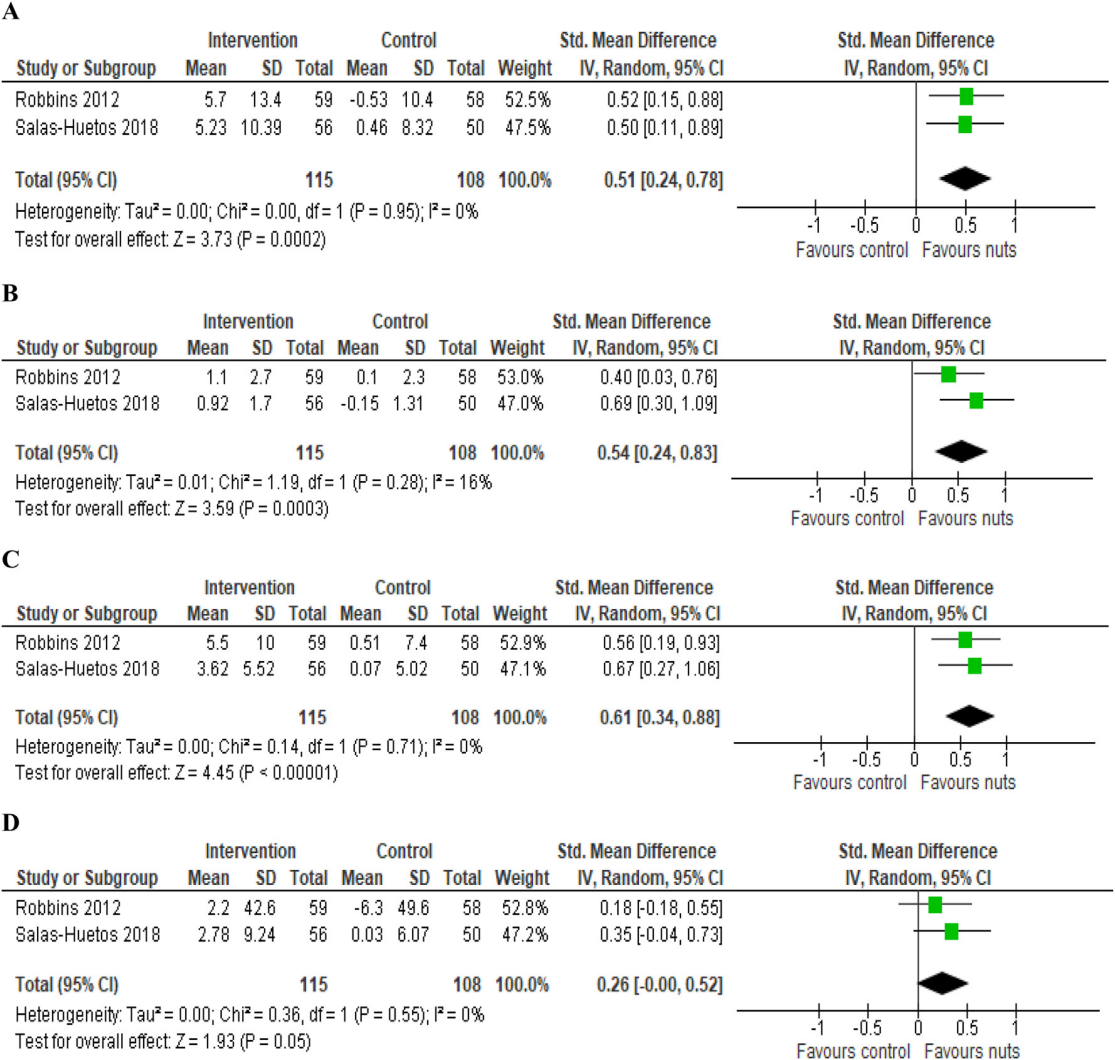
Effect of nut consumption on A) sperm total motility (% progressive plus nonprogressive); B) sperm morphology (% normal forms); C) sperm vitality (%); and D) sperm concentration (x 106/mL) in healthy males. Standardized mean difference (95% CI) shown for individual and pooled trials.

Given the homogeneous study populations, interventions, outcomes, and risk of bias observed between the 2 RCTs included in this review, we conducted a meta-analysis considering conventional semen parameters. Both the interventions with walnuts (75 g/d) [32] and mixed nuts (60 g/d) [33] resulted in significant increases in semen total motility (SMD in % progressive plus nonprogressive: 0.51; 95% CI: 0.24, 0.78; *P* < 0.001) (Figure 3A), morphology (SMD in % normal forms: 0.54; 95% CI: 0.24, 0.83; *P* < 0.001) (Figure 3B) and vitality (SMD as %: 0.61; 95% CI: 0.34, 0.88; *P* < 0.001) (Figure 3C) in comparison to control group, whereas no differences were observed for sperm concentration (SMD as × 10^6^/ml): 0.26; 95% CI: -0.00, 0.52; *P* = 0.05) (Figure 3D). Salas-Huetos et al. [33] further reported that the addition of mixed nuts to the usual diet for 14 wk significantly increased total sperm count (median change: 4.45 × 10^6^/ml, *P* = 0.002 for treatment effect), reduced sperm DNA fragmentation (*P* < 0.001 for treatment effect) and the expression of hsa-miR-34b-3p (*P* = 0.036 for treatment effect). In that study, no significant effect of the intervention was observed for sperm volume, pH, ROS production, global DNA methylation, and chromosome stability. Robbins et al. [32] also reported that the consumption of 75 g of walnuts daily reduced aneuploidy within the treatment group after 12 wk (*P* = 0.003), even though there was no difference between study groups at the end of the intervention. Further, the consumption of walnuts did not have significant effect on blood hormone concentrations. As the 2 observational studies had different study designs (longitudinal cohort and case-control), it was not appropriate to combine their results in a meta-analysis.

## Discussion

**FIGURE 4:**
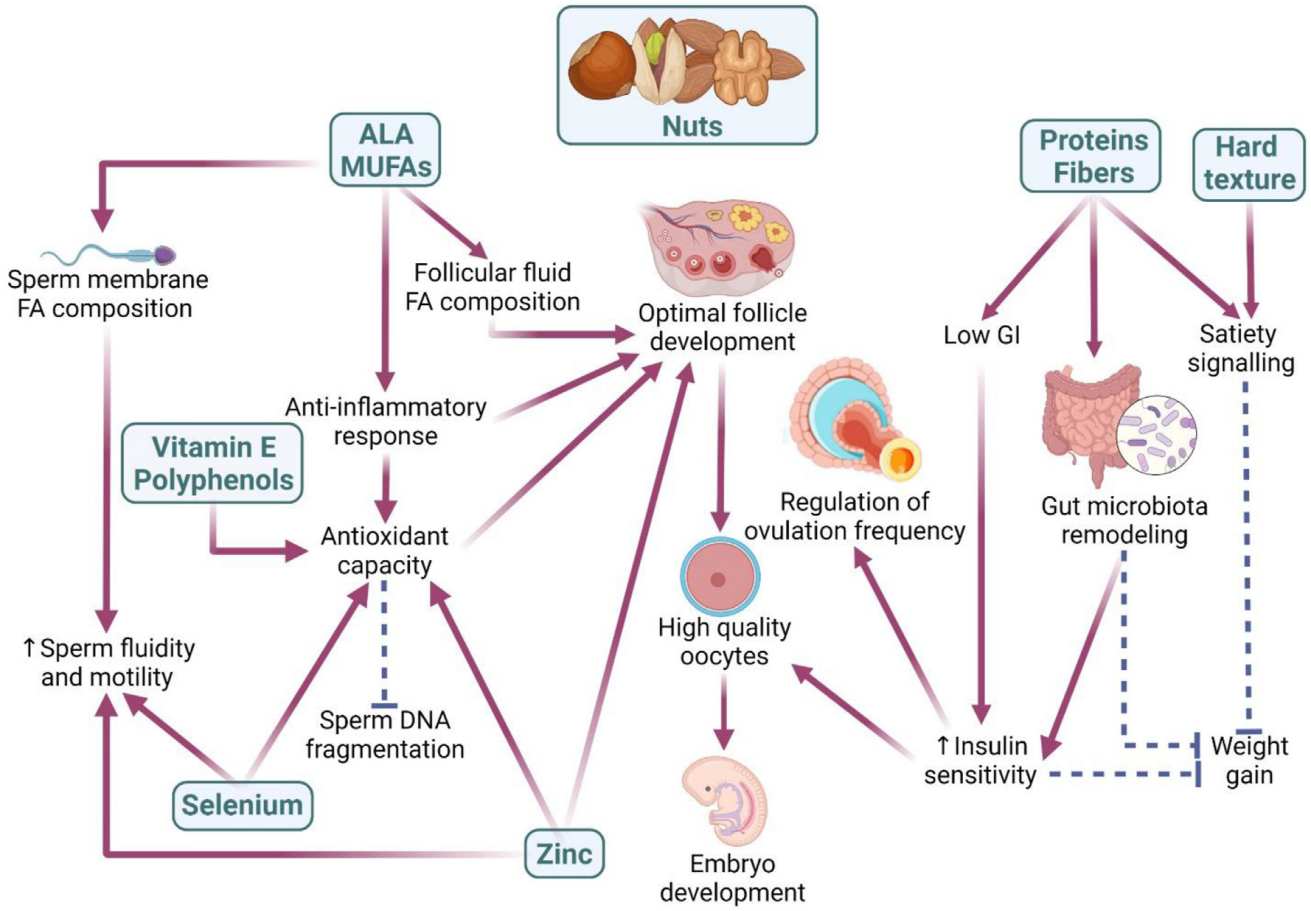
Hypothesis diagram for the role of nuts in fertility: Nuts are highly concentrated in ALA and MUFAs, which positively regulate the fatty acid concentrations in the follicular fluid (within the follicle) and sperm membrane and enhance the anti-inflammatory pathway, which increases the antioxidant capacity. Selenium, zinc, vitamin E and polyphenols present in nuts also enhance the antioxidant capacity. Higher concentration of antioxidants in the follicular fluid correlates with production of high-quality oocytes, which ultimately contributes to successful embryo development. During spermatogenesis, the developing sperm cells are highly sensitive to oxidative stress in seminal fluid; thus, the higher concentration of antioxidants reduces sperm DNA fragmentation. The high concentration of proteins and fibers in nuts results in beneficial gut microbiota remodeling, enhancement of satiety signaling, and improved glucose tolerance, which are all associated with a decrease in weight gain despite the high energy concentration seen in nuts. The hard texture presented by nuts also contributes to satiety signaling. Increased insulin sensitivity caused by nuts is associated with higher quality oocytes and a regulation of ovulation frequency (in opposition to reduction of ovulation frequency observed in females with insulin resistance). Created with Biorender.com.

This systematic literature review and meta-analysis were designed to provide insight into research exploring the effects of nuts as a nutritional strategy to improve reproductive health. Despite the flexible inclusion criteria, which encompassed different study designs, only 4 papers were identified and included in our review. Two randomized studies targeting healthy males reported that the consumption of at least 2 serves of nuts per day as part of a Western-style diet improves semen parameters such as sperm vitality, motility, and morphology. In contrast, 2 nonrandomized studies involving participants with varied fertility status found no convincing evidence for the association between dietary nut consumption of ≤ 1 serving per day and markers of sperm quality (males) or rates of embryo implantation, clinical pregnancy, or live birth following ART (females and males). Given the limited findings in the literature, we here discuss possible mechanisms that explain the benefits of nuts to fertility (Figure 4).

Western-style diet is considered a type of unhealthy diet given the high presence of ultra-processed foods containing high concentrations of saturated fatty acids, salt, and refined and simple carbohydrates, along with low intake of fresh produce [36]. This diet is strongly associated with chronic diseases such as obesity and type 2 diabetes [37]. Further, evidence compiled in literature reviews demonstrates that Western diets have been associated with an overall decline in male reproduction health [38]; more specifically, diets rich in typical Western-diet foods such as processed meat, full-fat dairy products, sweets, and sugar-sweetened beverages have been linked to worse semen quality [39]. In females, Western diets were associated with high rates of miscarriages [40] and infertility due to hormonal alterations that contribute to ovulation impairment [36]. Despite the close association between the consumption of Western-style diets and negative health outcomes, global dietary trends clearly show a rise in the consumption of saturated fat, sweeteners, and refined grains, indicating a steady increase in the consumption of ultra-processed foods across the globe [38]. Therefore, dietary strategies that can effectively offset the negative impact of ultra-processed foods are of major importance.

Tree nuts have an optimal fatty acid profile due to a high concentration of MUFA and polyunsaturated fats and a low concentration of saturated fats. High concentration of MUFA in blood, which is highly present in hazelnuts, peanuts, and almonds, was associated with increased fecundability in normal-weight females with prior pregnancy loss in a prospective cohort [41]. Further, an observational study of over 18,000 females without fertility problems demonstrated that swapping 2% of energy intake from trans fatty acids with MUFA was associated with less than half of risk of ovulatory infertility [42]. If we consider a 2000 kcal standard diet, which would meet the nutritional needs of most adults, this swap represents the inclusion of ∼37g of walnuts (one of the least tree nuts concentrated in MUFA) or 7.5g of macadamia (one of the most concentrated sources of MUFA among tree nuts), which would supply around 4.5 g/MUFA (40 kcal). Even though it remains to be explored whether nut consumption can increase MUFA concentration significantly as a potential mechanism, we hypothesize that this can be a mechanism of action of nuts for positive fertility outcomes. Walnuts, in particular, are known to have a high concentration of alpha-linolenic acid (ALA), a plant-based omega-3 fatty acid [[43], [44], [45], [46]]. Higher omega-3 fatty acid concentration in the spermatozoa membrane is associated with better motility and membrane fluidity, characteristics required for effective fertilization [[47], [48], [49], [50]]. Evidence from randomized clinical trials has demonstrated that the lipid composition of the sperm membrane is highly influenced by dietary intake [49, 51, 52]. As such, supplementation with DHA alone or in conjunction with EPA at doses that ranged from 1 to 2 g/d to males with idiopathic oligoasthenoteratozoospermia [49] or other infertility conditions [51] resulted in a significant improvement in semen parameters, such as sperm total count, concentration, and motility. Additionally, reduced spermatozoa with DNA damage was seen in the trial conducted by Martínez-Soto et al. [51], which used a dose of 1.5g/d. The consumption of omega-3 fatty acids is known to increase the anti-inflammatory response, as opposed to omega-6 fatty acids. As such, nut intake is negatively associated with inflammatory markers [53, 54]. Further, higher consumption of omega-3 was demonstrated to be associated with improved fecundability, possibly via reduction of proinflammatory cytokines and adhesion molecules [41, 55]. Studies also indicate a positive regulation of the antioxidant response by omega-3 fatty acids, which is particularly important for spermatozoa given they are highly susceptible to peroxidative damage [49]. Randomized clinical trials demonstrated that supplementation with omega-3 to males with infertility increased their antioxidant capacity as measured by the activity of superoxide dismutase (SOD) and catalase [49], as well as the seminal antioxidant capacity [51]. Although more research is required to elucidate the mechanisms involved, animal studies have shown that part of this antioxidant-related effect of omega-3 fatty acids is related to their capacity to facilitate the translocation and activation of specific antioxidant response elements such as the Nrf2 [56, 57]. Research is more limited regarding the investigation of the role of omega-3 in female fertility, although it has also been suggested the existence of an association between fatty acids composition of follicular fluid and fertility capacity, as this is an important microenvironment for the development of oocytes [58]. In a study involving 100 females undergoing ART, analysis of the fatty acid composition of follicular fluid revealed that the amount of saturated and even polyunsaturated fatty acids was inversely associated with the number of mature oocytes. However, the amount of ALA was associated with an improvement in maturity of oocytes, which leads these oocytes to have a higher efficiency, size, and cell membrane integrity due to membrane fluidity [58].

Nuts are also known for their high content of compounds with antioxidant profile, such as polyphenols and vitamin E [59, 60]. Even though important variations in the concentration and types of bioactive compounds are observed across the different types of nuts, they overall offer a significant amount of these compounds. For instance, it is estimated that the consumption of 30 g/d of almonds and hazelnuts, which is aligned with current recommendations [61], provides up to 49% of the dietary recommendations for vitamin E [62]. Oxidative stress plays a fundamental role in the occurrence of infertility, as ROS affect many different physiological functions in the male and female reproductive tract [63, 64]. Oxidative stress leads to membrane lipid damage and inhibition of protein synthesis and ATP depletion, which impairs oocyte maturation, as well as decreases ovarian steroidogenesis, ovulation, implantation, blastocyst formation, luteolysis, and luteal maintenance during pregnancy. In males, oxidative stress is associated with lipid peroxidation of the sperm cell membrane, sperm DNA damage, and apoptosis [65, 66]. A frequent consequence of excessive ROS is sperm DNA fragmentation, which is correlated with abnormal sperm morphology and motility and impairs blastulation, implantation, and early embryo development [67]. In couples undergoing ART, sperm DNA fragmentation is associated with reduced likelihood of pregnancy and increased risk of miscarriage [68]. Even though research is inconsistent in demonstrating the benefits of antioxidant supplementation to improve fertility outcomes, mostly due to heterogeneous studies [69, 70], we hypothesize that one of the mechanisms driven by the consumption of nuts on fertility is through the improvement of the antioxidant system.

The different types of nuts also provide significant amounts of minerals. Of particular interest for fertility, Brazil nuts are the richest food source of selenium, necessary for fertilization and embryo development, as well as protection of sperm against oxidative stress. In that regard, selenium deficiency, which affects 1 in 7 people around the globe [71], is associated with idiopathic infertility, higher rates of miscarriage, preterm birth, decreased sperm motility, and fertilization capacity [72]. Several trials with selenium supplementation showed positive effects on sperm quality parameters; however, the findings are not consistent across the literature [73]. This is probably because selenium supplementation does not seem beneficial to selenium-replete populations, and thus, the inclusion of Brazil nuts in the diet would have higher potential to enhance fertility capacity among those with selenium deficiency. Zinc, also present in important concentrations in several types of nuts such as cashews, almonds, and pine nuts, plays a critical role in male fertility, as it is required for sperm maturation, motility, capacitation, and acrosomal exocytosis [74]. In females, zinc is essential for processes that regulate follicle development, oocyte maturation, and fertilization, highlighting the importance of this mineral in all the steps involved in normal fertility [75].

Low glycemic index diets improve several features of PCOS, such as hormonal profile, insulin resistance, blood lipids, and fertility, as reported by a meta-analysis of clinical trials [76)]. Insulin resistance seems to also be linked to male infertility, as evidenced by lower semen volumes and higher sperm DNA fragmentation and mitochondrial DNA damage rates in males with diabetes in comparison to controls [77]. Given that nuts are considered to have a low glycemic index due to their high concentration of proteins, fibers, and fatty acids, we can contemplate that regulation of blood glucose response is one of the mechanisms involved in the effects of nuts on fertility. Further, polyphenols present in nuts seem to inhibit enzymes required for carbohydrate digestion, such as alpha-glucosidase, which is reflected in lower postprandial blood glucose responses [78]. Also, there is suggestive evidence indicating that nuts have a prebiotic effect [79], which means that they can selectively stimulate the growth of beneficial bacterial species in the gut. By remodeling the gut microbiome, nuts as prebiotics can decrease inflammatory markers and improve blood glucose response [80]. A meta-analysis reported that treatment with probiotics or synbiotics, which positively shift the gut microbiome, is effective in decreasing markers of insulin resistance in females with PCOS [81]. Thus, the inclusion of nuts as part of a low glycemic index diet can be used as a helpful strategy to benefit particularly males and females with insulin resistance, and type 1 or type 2 diabetes who are trying to conceive.

Obesity is a major risk factor for infertility. Males with overweight/obesity present with worse sperm quantity/quality [82], obese females have higher risk of PCOS and disrupted hormonal profile with lower levels of LH and gonadotropin hormone-releasing hormone, which negatively reflect on fertility. Further, obesity is associated with a reduction in the success of fertility treatments [83]. Despite their high energy density, nuts do not seem to be associated with overweight or obesity, as reported by a meta-analysis of 6 prospective cohort studies and 86 randomized clinical trials [84]. Paradoxically, higher consumption of nuts was significantly associated with a decrease in body weight and body fat, indicating a protective effect against adiposity [84]. The main explanations rely on the fact that nuts promote satiety due to their high concentration of fiber and proteins, as well as their physical structure, which requires effective mastication that triggers satiating signaling mechanisms and reduces bioavailability of macronutrients and, hence, energy. Such effects seem to be more important when nuts are consumed as a snack instead of being combined with other foods in a meal [85]. Further, their high concentration of unsaturated fatty acids is hypothesized to contribute to higher thermogenesis [86]; however, evidence is inconsistent in demonstrating that energy expenditure increases due to nut consumption [85].

This systematic review and meta-analysis were limited to only 4 studies available in the literature, which flags the necessity for other studies to better identify the potential benefits of introducing nuts into the diet to improve fertility outcomes. Although the 2 RCTs reported positive effects of ≥2 servings of nuts/d in sperm parameters of healthy males, the 2 observational studies did not find significant associations between nut consumption and fertility health. Significant differences between the RCTs and the observational studies should be highlighted in order to understand these discrepancies. First, whereas the RCTs provided at least 2 servings of nuts/d, the observational studies were conducted in populations with a rather low nut consumption (one reported an average intake of 0.9 servings of total nuts/d [34] and the other one reported a median consumption between 8 and 12 g/d) [35]. Second, it is important to note the differences in the study populations included in the RCTs versus the observational studies: whereas the RCTs recruited healthy males, the prospective study by Salas-Huetos [34] recruited males and females with fertility issues. Further, even though the control males in the study by Yorusun [35] had normal sperm quality, they may have had other sperm abnormalities that were not measured in the study (for example, sperm DNA fragmentation). Also, whereas the prospective study design and the statistical analysis that adjusted for diet quality in the study conducted by Salas-Huetos [34] minimizes reverse causality, the study conducted by Yorusun [35] did not consider any dietary or behavioral factors in the data analysis, even though it is known that semen quality may be affected by other aspects such as diet quality, BMI and smoking status. Factors affecting risk of bias in the included studies must also be considered when interpreting the results of this review. Three of the 4 studies did not disclose whether outcome assessors or statisticians were blinded, potentially introducing a source of observer or detection bias. However, this type of bias was unlikely in the included studies as laboratory staff assessing sperm quality were unlikely to be aware of participant dietary intakes. Failure to adjust for important covariates such as participant age and BMI during statistical analysis may have introduced confounding bias into one study [35] which was determined to have a serious risk of bias, so results should be interpreted with caution. All 4 studies assessed semen parameters which, despite being a predictor of male fertility, do not necessarily reflect patient-important fertility outcomes such as pregnancy and live birth. Further, the 2 randomized studies t provided participants with ≥ 2 servings of nuts per day both involved healthy males, which limits the extrapolation of the findings to those who experience subfertility or infertility and are, therefore, the most interested in the effects of dietary strategies for fertility health. Additionally, given that infertility due to female factors is impactful and can range from 30 to 80% [4], more studies targeting females are necessary to elucidate the effects of nuts on reproductive success.

## Conclusions

Due to their nutritional profile, nuts seem to have the potential to promote successful reproductive outcomes. Our meta-analysis shows that including ≥ 2 servings of nuts daily as part of a Western-style diet in healthy males improves sperm parameters, which are predictors of male fertility. Nonetheless, the number of high-quality RCTs involving nut intake as a strategy for infertility treatment is scarce and limited to males, even though females’s infertility problems affect a significant proportion of couples’ ability to conceive. Therefore, given that infertility is considered a major health subject and now more than ever people seek more natural and affordable alternatives to deal with it, we advocate for future studies that target different populations to include females and males who are having difficulties conceiving and have pregnancy as primary outcome. Given the differences in the nutritional composition of the different types of nuts, future studies should also consider combining different nuts to investigate potential synergistic effects regarding the positive effects on fertility outcomes.

## Data Availability

Corresponding authors will provide data and other materials used in this review upon reasonable request.
