# Supplementary material for: Nut Consumption and Fertility: a Systematic Review and Meta-Analysis
Source: Adv Nutr. 2023 Nov 17;15(1):100153. doi: 10.1016/j.advnut.2023.100153 (PMC10704322; doi:10.1016/j.advnut.2023.100153)
Supplement: Multimedia component 1 [file mmc1.docx]

**Cardoso BR et al. Nut consumption and fertility: a systematic review and meta-analysis**

**Supplemental Table S1.** Example of database search strategy used (Ovid MEDLINE on 30/06/2023)

| **#** | **Search term** |
| --- | --- |
| 1 | exp Adult/ |
| 2 | exp Diet/ |
| 3 | exp Nuts/ |
| 4 | (Juglans or Walnut*).mp. |
| 5 | (Prunus dulcis or Almond*).mp. |
| 6 | (Anacardium or Cashew*).mp. |
| 7 | (Corylus or Hazelnut*).mp. |
| 8 | (Pistacia or Pistachio*).mp. |
| 9 | (Carya or Pecan*).mp. |
| 10 | (Arachis or Peanut*).mp. |
| 11 | (Pinus or Pine nut*).mp. |
| 12 | (Bertholletia or Brazil nut*).mp. |
| 13 | 2 or 3 or 4 or 5 or 6 or 7 or 8 or 9 or 10 or 11 or 12 |
| 14 | exp Pregnancy/ |
| 15 | pregnan*.mp. |
| 16 | live birth*.mp |
| 17 | exp Fertilization/ |
| 18 | exp Fertility/ |
| 19 | exp Infertility/ |
| 20 | exp Infertility, Male/ |
| 21 | exp Infertility, Female/ |
| 22 | fecundity.mp. |
| 23 | fecundability.mp. |
| 24 | subfertility.mp. |
| 25 | sperm dysfunction*.mp. |
| 26 | (sperm or sperm DNA damage or sperm DNA integrity or sperm parameters or semen parameters or sperm damage or sperm quality or semen quality or sperm volume or sperm concentration or sperm motility).mp. |
| 27 | (varicocele or asthenozoospermia or oligozoospermia or oligospermia or  oligoasthenozoospermia or oligoasthenospermia or oligoasthenoteratozoospermia or  teratozoospermia).mp. |
| 28 | (still birth or stillbirth or still born or stillborn or miscarr*).mp. |
| 29 | abortion.mp. |
| 30 | (embryo* or embryo morphology or oocyte*).mp. |
| 31 | 14 or 15 or 16 or 17 or 18 or 19 or 20 or 21 or 22 or 23 or 24 or 25 or 26 or 27 or 28 or 29 or 30 |
| 32 | 1 and 13 and 31 |
